# Inhomogeneity of immune cell composition in the synovial sublining: linear mixed modelling indicates differences in distribution and spatial decline of CD68+ macrophages in osteoarthritis and rheumatoid arthritis

**DOI:** 10.1186/s13075-016-1057-3

**Published:** 2016-07-16

**Authors:** Johanna Mucke, Annika Hoyer, Ralph Brinks, Ellen Bleck, Thomas Pauly, Matthias Schneider, Stefan Vordenbäumen

**Affiliations:** Hiller Research Center Rheumatology at University Hospital Düsseldorf, Medical Faculty, Heinrich-Heine-University, Merowingerplatz 1a, 40225 Düsseldorf, Germany; German Diabetes Center, Institute for Biometry and Epidemiology, Düsseldorf, Germany; Department Orthopaedics, River Rhein Center for Rheumatology at St. Elisabeth Hospital, Meerbusch-Lank, Germany

**Keywords:** Rheumatoid arthritis, Osteoarthritis, Sublining layer, Macrophages, CD68, Synovitis score

## Abstract

**Background:**

Inhomogeneity of immune cell distribution in the synovial sublining layer was analyzed in order to improve our mechanistic understanding of synovial inflammation and explore potential refinements for histological biomarkers in rheumatoid arthritis (RA) and osteoarthritis (OA).

**Methods:**

Synovial tissue of 20 patients (11 RA, 9 OA) was immunohistochemically stained for macrophages (CD68), synovial fibroblasts (CD55), T cells (CD3), plasma cells (CD38), endothelial cells (vWF) and mast cells (MCT). The synovial sublining layer was divided into predefined adjacent zones and fractions of the stained area (SA) were determined by digital image analysis for each cell marker.

**Results:**

Distribution of CD68, CD55, CD38 and MCT staining of the sublining area was heterogeneous (Friedman ANOVA *p* < 0.05). The highest expression for all markers was observed in the upper layer close to the lining layer with a decrease in the middle and lower sublining. The SA of CD68, CD55 and CD38 was significantly higher in all layers of RA tissue compared to OA (*p* < 0.05), except the CD38 fraction of the lower sublining. Based on receiver operating characteristics analysis, CD68 SA of the total sublining resulted in the highest area under the curve (AUC 0.944, CI 95 % 0.844–1.0, *p* = 0.001) followed by CD68 in the upper and middle layer respectively (both AUC 0.933, CI 95 % 0.816–1.0, *p* = 0.001) in both RA and OA. Linear mixed modelling confirmed significant differences in the SA of sublining CD68 between OA and RA (*p* = 0.0042) with a higher concentration of CD68+ towards the lining layer and more rapid decline towards the periphery of the sublining in RA compared to OA (*p* = 0.0022).

**Conclusions:**

Immune cells are inhomogeneously distributed within the sublining layer. RA and OA tissue display differences in the number of CD68 macrophages and differences in CD68 decline within the synovial sublining.

## Background

Histological analysis of the synovial membrane is a powerful tool for the investigation of pathological changes in rheumatoid arthritis (RA) in order to elucidate the pathogenic mechanisms involved in the disease [1]. In addition, the assessment of synovial biomarkers is quite useful in dose-finding studies, for the stratification of patient groups, and to identify new therapeutic targets [2]. Although not part of the clinical daily routine, the use of synovial biopsies in certain clinical situations is unquestioned [3–5]. For instance, CD68-positive macrophages in the sublining layer have repeatedly been shown to be one of the best activity markers for RA [6, 7]. Besides macrophages, further cells are of major interest in synovial biopsies: synovial fibroblasts are considered key players in the pathogenesis of rheumatoid arthritis [8]. T cells are major components of inflammatory infiltrates and trigger autoimmunity in cooperation with antibody-producing plasma cells [9–11]. Mast cells have been identified to modulate B cells and produce proinflammatory cytokines in RA [12, 13] whereas endothelial cells function as a marker for increased angiogenesis in inflamed tissue [14].

Although the synovial sublining is generally considered as a whole, we consistently noted inhomogeneous distribution of immune cells, particularly prominent under pathological conditions within this particular compartment of the synovium. A more precise definition of the relevant areas within the sublining layer might improve our pathophysiologic understanding of inflammatory joint diseases and potentially lead to improved diagnostic usage of synovial biopsies. Thus, we set out to analyze histological features and the cellular composition of the sublining layer in more detail.

## Methods

### Patients and synovial sampling

Synovial tissue was obtained from a total of 20 patients (11 RA, 9 OA) who underwent synovectomy (elbow (*n* = 1), wrist (1), shoulder (1) or total joint replacement (11 hips, 6 knees)) at the Department of Orthopaedics at the River Rhein Center for Rheumatology, St. Elisabeth Hospital, Meerbusch-Lank, Germany. All patients diagnosed with RA fulfilled the 2010 American College of Rheumatology criteria for RA. Osteoarthritis (OA) was diagnosed based on the ACR criteria for knee or hip OA [15, 16]. All patients gave their full informed consent. The samples were taken under visual control from macroscopically inflamed areas, were immediately snap frozen in tissue-TEK (Sakura Finetek Germany, Staufen, Germany) and stored at −80° until further processing.

### Histology and immunohistochemistry

Seven-micron sections were obtained from the snap-frozen tissue and fixed for 10 minutes in 3 % paraformaldehyde in phosphate-buffered saline (PBS). After conventional hematoxylin and eosin (H&E) staining (Merck, Darmstadt, Germany), synovial morphology was evaluated for tissue quality and the presence of a continuous lining layer. The sections were used for the determination of the synovitis score according to Krenn [17], which is a semi-quantitative 4-point sum score assessing the synovial lining layer hypertrophy, inflammatory infiltrate and cellular density of resident cells. For immunohistochemistry, parallel sections were incubated with primary monoclonal mouse antibodies against CD68, mast cell tryptase (MCT), CD15, CD19, CD56 (all Dako, Glostrup, Denmark), CD55 (SouthernBiotech, Birmingham, AL, USA), CD3, CD38, von Willebrand factor (vWF), CD83 (all BD Biosciences, San Jose, CA, USA), IgG1 as isotype control (Dako, Glostrup, Denmark) and secondary antibody of the Dako Real Detection System (Dako, Glostrup, Denmark), according to the manufacturer’s instructions. In three cases tissue quantity was insufficient for sublining layer analysis of single antibodies (1 × CD68 (RA), 2 × MCT (RA, OA)).

### Imaging and calculation of stained areas

Sections were photographed at × 200 magnification (Axioskop 2 plus: Carl Zeiss, Jena, Germany; Nikon DS Vi 1: Nikon, Düsseldorf, Germany) and stored in TIF format (resolution of 1600 × 1200, 96 dpi) (Image acquisition software: NIS-Elements F, Nikon). Rectangular regions of interest (ROI) of 500 × 250 pixels (661.5 μm × 330.5 μm) size were created using ImageJ [18] and the upper sublining ROI was placed adjacent to the lining layer with the lower layer at greatest distance from the synovial surface. ROIs for the middle and lower layer were set contiguously in a row. Visual inspection of all tissues preceded the definition of the ROIs’ size of 500 × 250 pixels, which was considered suitable to delineate each layer separately without including parts of the opposite sublining area, especially critical in villous formations of RA tissue. The lumina of blood vessels within the selected regions were delineated and subtracted from the respective layer area still including respective endothelial cells in the analysis. Images were then thresholded to highlight the stained areas but not the respective isotype controls. After converting the image into a binary image, the highlighted section was measured and presented as a fraction of the selected region. For linear mixed model analysis the three ROIs were divided in half to create six equally sized ROIs. To obtain representative results, measurements were made from three different regions of each sample and mean values were used for statistical analysis.

### Statistical analyses

For continuous scales data are given as mean ± standard deviation (SD), ordinal data such as the synovitis score is presented as median and 1st quartile to 3rd quartile (interquartile range, IQR). Student’s *t* test for independent samples and Mann–Whitney *U* test were used to compare the two groups as appropriate. Analysis of the different layers was carried out with Friedman’s two-way analysis of variance (ANOVA) and Dunn’s post hoc test. Correlations between the synovitis score and the stained areas were calculated according to Spearman. Receiver operating characteristics (ROC) analysis with calculation of the area under the cure (AUC) was used to examine the diagnostic value of the evaluated cell markers. Aforementioned statistical analyses were carried out using IBM SPSS statistics (IBM Corp., Armonk, NY, USA) at a significance level of α = 0.05. For comparison of the decline in CD68+ staining between OA and RA, we applied a linear mixed model (LMM) with random intercept for the CD68+ concentration with following independent variables: distance of the ROI, disease status and interaction between distance and disease status. For the LMM we used the function PROC MIXED of SAS 9.3 (SAS Institute Inc., Cary, NC, USA).

## Results

### Patients’ demographics and clinical features

Eleven patients with RA (nine female, aged 63.5 ± 10.6 years) and nine with OA (six female, aged 69.4 ± 11.1 years) were included in this study. Of the RA patients three had synovectomy of shoulder, hand and elbow, respectively. Five underwent total hip replacement and three had a total knee replacement. OA tissue was obtained from six patients undergoing total hip replacement and three cases of total knee replacement. Demographic and clinical data is summarized in Table 1.

**Table 1:** Demographic and clinical features

	All patients	RA	OA	RA vs. OA
	*n* = 20	*n* = 11	*n* = 9	*p* values
Age at surgery, yrs (±SD)	66.2 (±10.9)	63.5 (±10.6)	69.4 (±11.1)	0.233
Female, n (%)	14 (75.0)	9 (81.8)	6 (66.7)	
CRP, mg/dl (±SD)	2.5 (±2.7)	3.7 (±3.2)	1.2 (±1.0)	**0.001**
Leucocytes,/μl (±SD)	8570.0 (±4784.4)	10,654.5 (±5639.0)	6022.2 (±1157.3)	**0.001**
RF, IU/ml (±SD)	71.5 (±156.9)	126.3 (±198.5)	4.4 (2.4)	**<0.001**
ESR, mm/h, (±SD)	22.2 (±20.9)	29.4 (±23.3)	14.1 (15.3)	0.079

Comparison by Student’s *t* test, significant results are printed in bold

*RA* rheumatoid arthritis, *OA* osteoarthritis, *SD* standard deviation, *CRP* C-reactive protein, *RF* rheumatoid factor, *ESR* erythrocyte sedimentation rate

### Synovitis score (H&E staining)

On histological analysis of H&E-stained sections, the median synovitis score was 6 (interquartile range (IQR) 5–7) in RA patients and 3 (IQR 1.5–5) in the OA group (*p* = 0.002). The RA group showed significantly higher numbers for all three subscores, e.g. lining layer, inflammatory infiltrate, and cellular density (Table 2).

**Table 2:** Synovitis score

	All patients	RA	OA	RA vs. OA
	*n* = 20	*n* = 11	*n* = 9	*p* values
Synovitis score^a^, median (IQR)	5 (3–6)	6 (5–7)	3 (1.5–5)	**0.002**
Lining layer hypertrophy	1.5 (1–2)	2 (1–3)	1 (0–1.5)	0.025
Inflammatory infiltrate	1.5 (1–3)	3 (1–3)	1 (0.5–1.5)	**0.007**
Cellular density	2 (1–2)	2 (2–2)	1 (0.5–1.5)	**0.002**

Comparison by Student’s *t* test, significant results are printed in bold

*RA* rheumatoid arthritis, *OA* osteoarthritis, *IQR* interquartile range

^a^Synovitis score according to Krenn and colleagues [17]

**Fig. 1:**
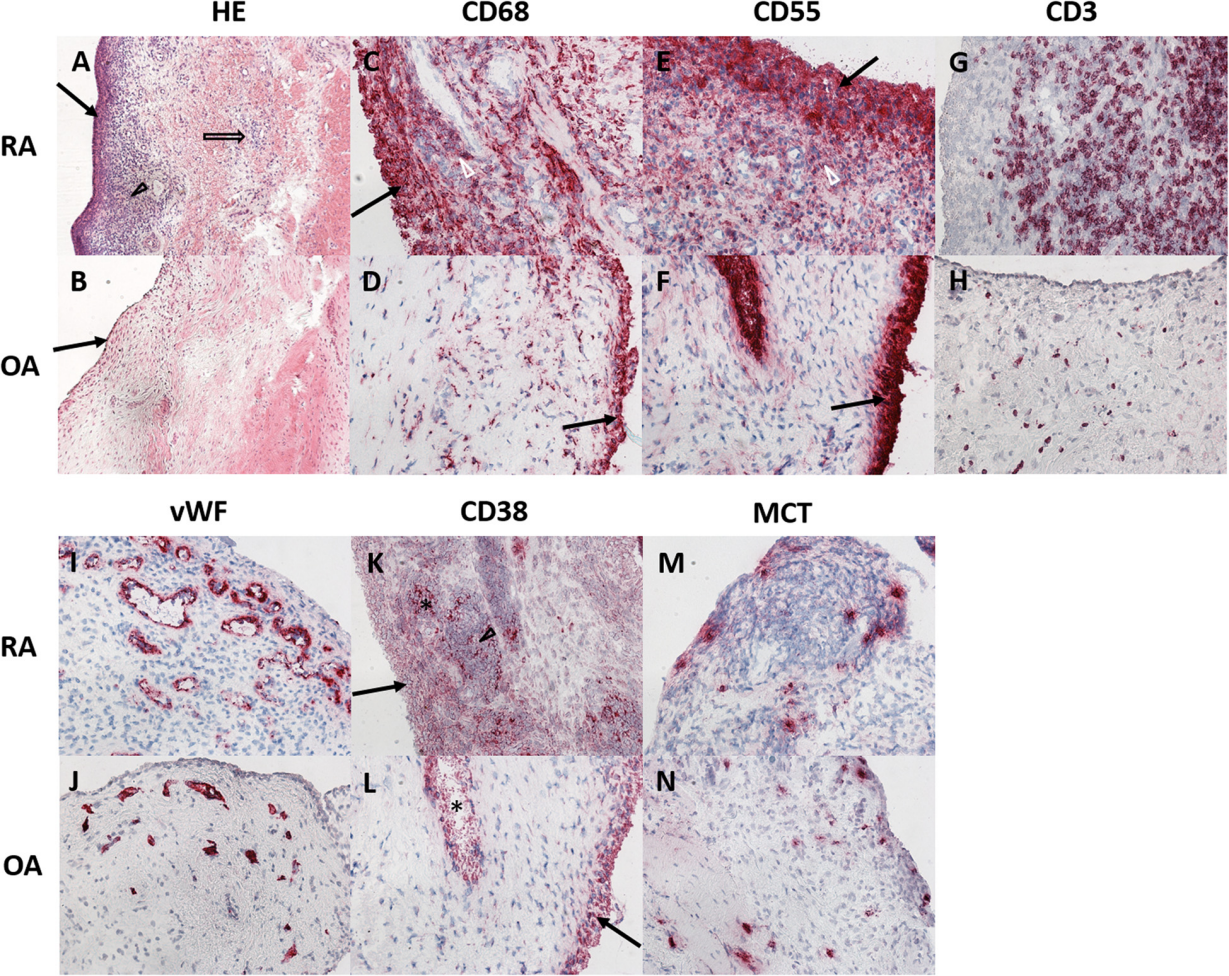
Typical histologic and immunohistochemical staining patterns of RA and OA synovial tissue. H&E staining reveals an enlarged synovial lining layer (*black arrows*), an increased cellular density (*hollow arrow*) and inflammatory infiltrates (*arrowhead*) in RA tissue, the findings are less marked in OA tissue. CD68 and CD55 expression is predominant in the lining layer (*black arrow*) and upper sublining (*white arrowhead*) adjacent to the lining, again more pronounced in RA compared to OA, whereas CD3+ T cells are distributed equally within the sublining. CD38 expression is observed in the lining layer (*black arrow*) and vascular structures (^*^) as well as in lymphocytic infiltrates (*arrowhead*). vWF and MCT staining is also more pronounced within the upper lining, although the difference between RA and OA is only mild

Next, we were interested to determine if the synovitis score as a measure of inflammatory activity in the entire synovial layer is reflected by individual cellular markers within the sublining layer. Correlation analyses revealed a moderate to high correlation for the total stained area of CD68, CD3 and CD55 and the total synovitis score with its subscores in all patients (RA and OA) except CD55 and the cellular density. CD38 and MCT total stained area did not correlate with the synovitis score, and vWF showed moderate correlation only with the subscore cellular density (Table 3). Typical histological findings of RA and OA are exemplified in Fig. 1.

**Table 3:** Correlation between the total stained area of the synovial sublining and the synovitis score

	Synovitis score^a^	Lining layer hypertrophy	Inflammatory infiltrate	Cellular density
CD68	**0.706 (** ***p*** **= 0.001)**	**0.554 (** ***p*** **= 0.014)**	**0.604 (** ***p*** **= 0.006)**	**0.576 (** ***p*** **= 0.010)**
CD3	**0.852 (** ***p*** **< 0.001)**	**0.798 (** ***p*** **< 0.001)**	**0.757 (** ***p*** **< 0.001)**	**0.601 (** ***p*** **= 0.005)**
CD55	**0.651 (** ***p*** **= 0.002)**	**0.622 (** ***p*** **= 0.003)**	**0.668 (** ***p*** **= 0.001)**	0.428 (*p* = 0.060)
CD38	0.245 (*p* = 0.298)	0.122 (*p* = 0.608)	0.154 (*p* = 0.518)	0.419 (*p* = 0.066)
vWF	0.437 (*p* = 0.054)	0.302 (*p* = 0.195)	0.344 (*p* = 0.138)	**0.576 (** ***p*** **= 0.008)**
MCT	0.083 (*p* = 0.743)	0.362 (*p* = 0.140)	−0.071 (*p* = 0.780)	0.008 (*p* = 0.974)

Correlations according to Spearman, significant correlations are printed in bold

*CD68* macrophages, *CD3* T cells, *CD55* synovial fibroblasts, *CD38* plasma cells, *vWF* von Willebrand factor, *MCT* mast cell tryptase

^a^Synovitis score according to Krenn and colleagues [17]

### Immune cells are inhomogeneously distributed within the sublining layer

**Fig. 2:**
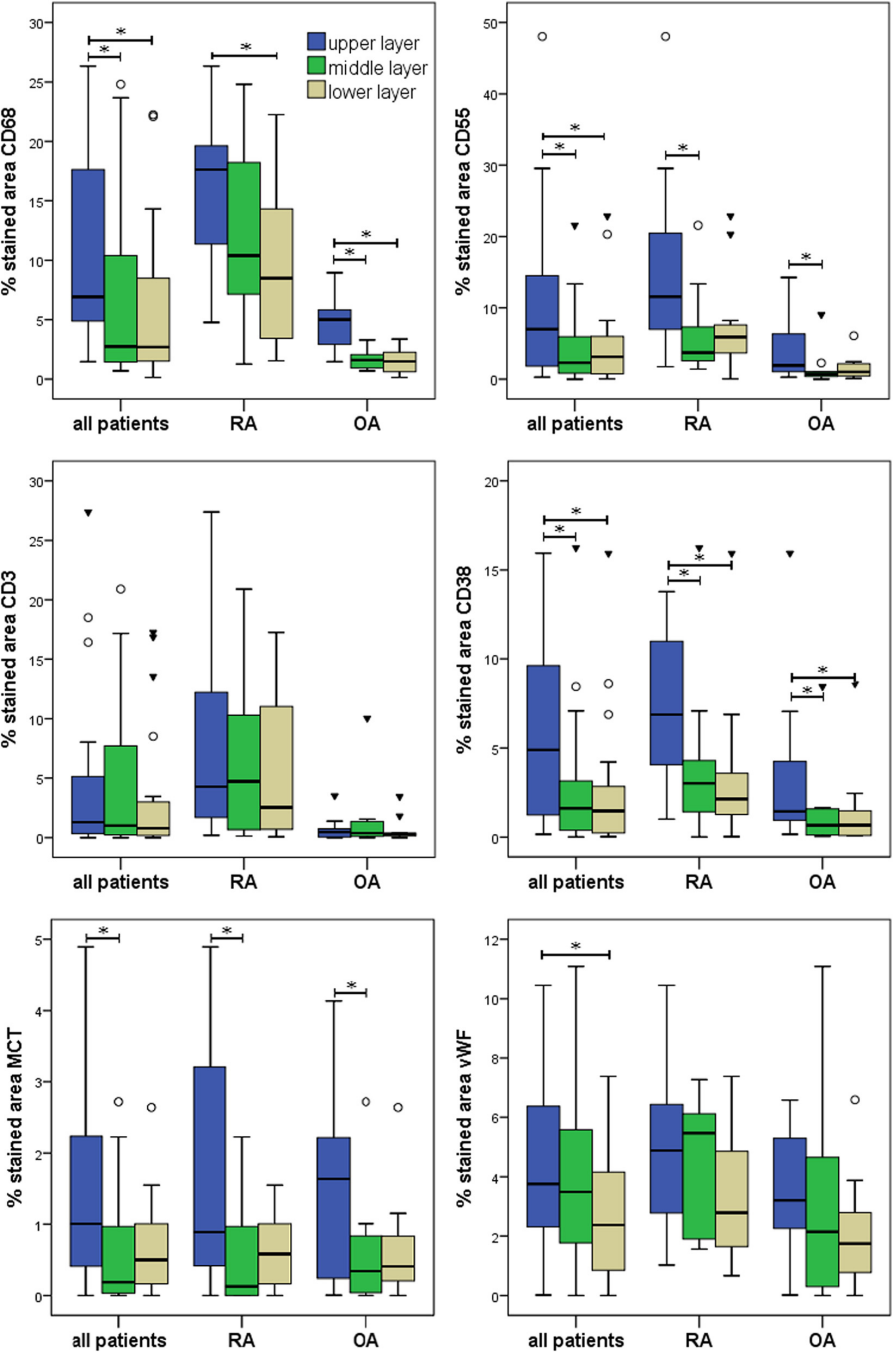
Differences within the sublining layer for expression of CD68, CD55, CD3, CD38 and MCT in all patients and patients with RA and OA respectively. Expression of cellular markers was highest in the upper sublining adjacent to the lining layer (*blue*), with a decrease towards the middle (*green*) and lower (*fawn*) layers within the deeper synovium (except CD3). ^*^Statistically significant; ^○^outliers; ^▼^extremes

In order to assess cellular distribution within the sublining layer, immunohistochemistry was applied to stain for macrophages (CD68), synovial fibroblasts (CD55), T cells (CD3), plasma cells (CD38), endothelial cells (vWF) and mast cells (MCT) (Fig. 1). The fraction of stained area was determined by digital image analysis in three predefined zones of the sublining layer with the upper layer closest to the lining layer and the lower layer representing the deeper sublining. While expression of CD68, CD3, CD55, vWF and CD38 could be visualized in all cases, MCT was abundant in three tissues (two RA, one OA). Analysis revealed an inhomogeneous distribution of CD68-, CD55-, CD38-, and MCT-positive cells (*p* < 0.05 according to Friedman two-way ANOVA). Staining of CD19+ B cells, CD15+ granulocytes, CD56+ natural killer cells and CD83+ dendritic cells was discontinued due to very low expression in both RA and OA tissue. Details on inhomogeneity of distinct immune cells within the sublining layer are given in Fig. 2.

### The percentage of stained area of CD68, CD3, CD55 and MCT differs significantly between RA and OA

We then set out to compare cell marker expression between RA and OA. These analyses revealed significant differences between all sublining layers with consistently higher percentages of staining in RA tissue for the three parameters CD68, CD55 and CD38. Typical staining patterns in RA and OA are shown in Fig. 2. Results of the comparison of RA and OA are summarized in Table 4.

**Table 4:** Mean percentage of stained area in the synovial sublining

		All patients	RA	OA	Mann–Whitney *U*
		(mean ± SD)	(mean ± SD)	(mean ± SD)	(*p* value)
CD68	Upper	10.52 (±7.75)	15.89 (±6.88)	4.55 (±2.42)	**0.001**
	Middle	7.12 (±7.82)	12.04 (±8.05)	1.66 (±0.89)	**0.001**
	Lower	5.92 (±6.92)	9.85 (±7.66)	1.56 (±1.06)	**0.001**
	Total area	7.85 (±7.33)	12.59 (±7.30)	2.59 (±1.34)	**<0.001**
CD3	Upper	4.79 (±7.74)	8.06 (±8.88)	0.79 (±1.11)	**0.006**
	Middle	4.38 (±6.27)	6.68 (±7.32)	1.58 (±3.22)	0.056
	Lower	3.56 (±5.70)	5.86 (±6.91)	0.75 (±1.15)	0.095
	Total area	4.24 (±6.05)	6.86 (±7.09)	1.04 (±1.78)	**0.012**
CD55	Upper	10.80 (±11.93)	15.81 (±13.61)	4.67 (±5.45)	**0.012**
	Middle	4.29 (±5.40)	6.46 (±6.11)	1.64 (±2.86)	**0.002**
	Lower	5.00 (±6.20)	7.73 (±7.21)	1.66 (±1.88)	**0.007**
	Total area	6.70 (±6.59)	10.00 (±7.00)	2.66 (2.92)	**0.002**
CD38	Upper	5.75 (±4.95)	7.43 (±4.34)	3.68 (±5.10)	**0.046**
	Middle	2.86 (±3.94)	3.97 (±4.55)	1.50 (±2.68)	**0.038**
	Lower	2.74 (±3.85)	3.60 (±4.52)	1.69 (±2.73)	0.175
	Total area	3.78 (±4.01)	5.00 (±4.16)	2.29 (±3.47)	**0.046**
vWF	Upper	4.19 (±2.67)	4.87 (±2.77)	3.37 (±2.43)	0.295
	Middle	3.84 (±2.92)	4.52 (±2.29)	3.02 (±3.51)	0.175
	Lower	2.88 (±2.28)	3.46 (±2.39)	2.18 (±2.06)	0.175
	Total area	3.64 (±2.17)	4.28 (±1.94)	2.86 (±2.30)	0.152
MCT	Upper	1.53 (±1.53)	1.53 (±1.68)	1.54 (±1.42)	0.897
	Middle	0.58 (±0.79)	0.53 (±0.73)	0.64 (±0.91)	0.696
	Lower	0.67 (±0.70)	0.66 (±0.59)	0.69 (±0.86)	0.897
	Total area	0.93 (±0.90)	0.90 (±0.85)	0.96 (±1.01)	0.829

Comparison of rheumatoid arthritis (RA) and osteoarthritis (OA) by Mann–Whitney *U*, significant results are shown in bold. Upper layer adjacent to lining layer; lower layer with greatest distance from lining layer within the deeper synovium

*RA* rheumatoid arthritis, *OA* osteoarthritis, *SD* standard deviation, *CD68* macrophages, *CD3* T cells, *CD55* synovial fibroblasts, *CD3*8 plasma cells*, vWF* von Willebrand factor, *MCT* mast cell tryptase

### CD68 remains the best parameter to distinguish RA from OA

In order to estimate the most reliable parameter for differentiation between RA and OA in the current study, receiver operating characteristics (ROC) analyses with determination of the area under the curve (AUC) were performed. CD68 total stained area within the sublining was identified as the most reliable marker to discriminate between RA and OA (AUC 0.94, CI 95 % 0.84–1.00, *p* = 0.001) followed by the CD68-stained area in the upper and middle sublining (both AUC 0.93, CI 95 % 0.82–1.00, *p* = 0.001). Furthermore, staining of CD3 upper layer (AUC 0.86, CI 95 % 0.69–1.00, *p* = 0.07) and CD55 middle layer (AUC 0.89, CI 95 % 0.71–1.00, *p* = 0.03) and the total stained area (CD3: AUC 0.83, CI 95 % 0.64–1.00, *p* = 0.014; CD55: AUC 0.89, CI 95 % 0.74–1.00, *p* = 0.03) provided considerable accuracy for RA tissue, whereas no difference was observed for CD38, vWF or MCT.

### Linear mixed modelling indicates significant differences in decline of CD68 staining within the synovial sublining between OA and RA

**Fig. 3:**
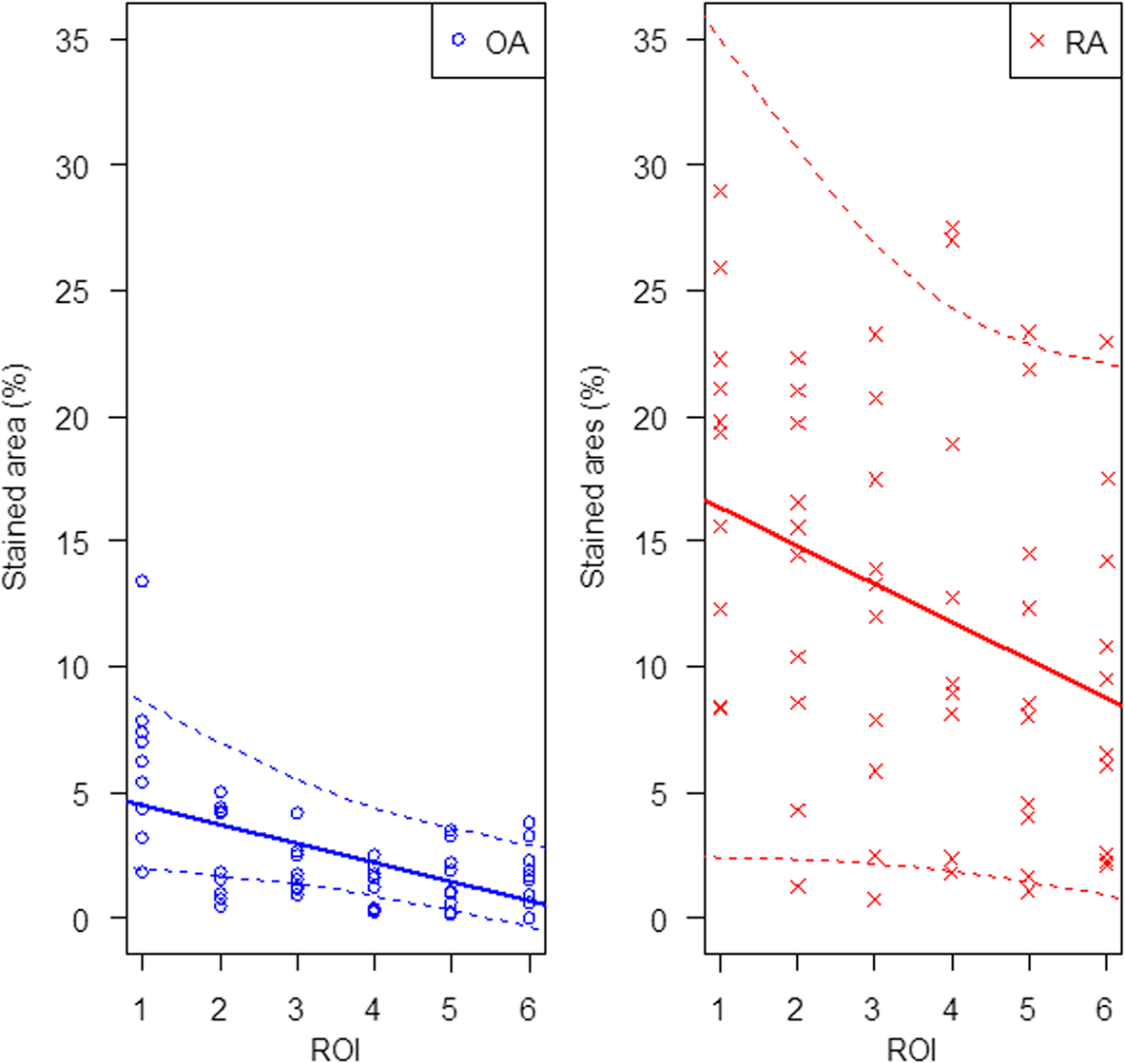
Linear mixed modelling indicates significant differences in decline of CD68 staining within the synovial sublining between OA and RA. RA shows a faster decline with distance from the lining layer from ROI 1 towards ROI 6 compared to OA

We set out to further specify the differences in CD68 expression between OA and RA by modelling the distribution of CD68-positive cells within the sublining layer. Three observations can be made: (1) in RA, the number of positive cells starts on higher level than in OA (*p* < 0.0001). (2) For both diseases, the number of positive cells decreases with growing distance from the lining layer (*p* < 0.0001). (3) The decrease is significantly stronger in RA compared to OA (*p* = 0.003). Details of the linear mixed model are outlined in Fig. 3 and Table 5.

**Table 5:** Linear mixed model of CD68+ macrophages spatial distribution within the synovial sublining: progressive decline in CD68+ macrophages with distance from the lining layer in OA and in RA

Effect	Disease	Estimate	Standard error	*p* value
Intercept		5.23	1.91	0.003
ROI distance		−0.75	0.20	<0.0001
Disease	OA	0		
Disease	RA	12.6	2.6	<0.0001
Interaction: ROI and disease	OA	0		
Interaction: ROI and disease	RA	−0.77	0.27	0.003

Estimates without standard error refer to the reference category

*ROI* region of interest, OA osteoarthritis, *RA* rheumatoid arthritis

## Discussion

The synovial membrane in patients with RA and OA has been subject to a broad variety of studies, which have substantially contributed to the elucidation of pathogenic mechanisms. So far, the lining layer has been intensively studied and histological features in RA such as hypertrophy and the accumulation of macrophages, fibroblasts and giant cells within the lining have been well described [19]. In this study, we focused on the sublining layer and the ongoing pathophysiological changes in this area since important observations have been made in this zone. In particular, CD68-positive sublining macrophages have been identified as a very potent biomarker: they reflect disease activity [20] and synovial inflammation in refined magnetic resonance imaging (MRI) procedures [21]. Most strikingly, changes in sublining CD68 macrophages are a potent biomarker for response to therapy across academic centres [6], and they are likely not liable to placebo effects [7]. This renders synovial biopsies a powerful tool in early- phase clinical studies [22]. These findings suggest that the synovial sublining may also play a substantial role in disease mechanisms of RA. However, the synovial sublining is ill-defined and our own circumstantial observations suggested that cellular distribution within this area may be inhomogeneous. In the present study, we partitioned the sublining layer and comprehensively analyzed immune cellular composition as this might lead to an improved understanding of disease mechanisms and potential future refinements in its use as a biomarker. We demonstrate a strikingly inhomogeneous distribution of most immune cells and fibroblasts within the sublining layer of both RA and OA tissue with a clear tendency of macrophages (CD68), synovial fibroblasts (CD55), plasma cells (CD38), mast cells (MCT) and endothelial cells (vWF) to accumulate in the upper sublining. Of note, we refrained from adjusting for multiple testing, because a low to moderate amount of statistical hypothesis was tested for the above markers, and because of concerns for overemphasizing the sensibility of *p* values [23]. However, as outlined in the tables, some borderline statistically significant findings would probably not have crossed the 5 % threshold in case of adjustments. Furthermore, we applied linear mixed modelling to the distribution of sublining CD68 cells in order to assess potential regularities in the distribution of macrophages with distance to the lining layer being the independent variable. The advantage of this particular model was a precise and accurate analysis of macrophage allocation since special focus was set on the distance to the lining taking into account the intra-patient correlations which were integrated into the statistical calculations [24]. We found a high accumulation of macrophages towards the lining layer and a fast decline in RA compared to OA. Since the lining layer faces the joint cavity, we assume that rather than the total CD68+ cells within the whole sublining layer, those in close proximity to the joint cavity are of foremost importance for the inflammatory joint reaction [25]. This is further supported by looking at the pathophysiological implications of CD68 homing: the increase of vWF expression reflects the early dysregulation of angiogenesis that occurs in inflammatory disorders [26] and is considered to be a prerequisite for immune cells to enter the synovial membrane [14, 26]. In RA, the process of angiogenesis and the subsequent recruitment of immune cells and synovial fibroblasts further results in the formation of pannus tissue producing inflammatory cytokines that lead to cartilage and bone destruction [27]. The close proximity of the respective immune cells to the lining layer and thus the surface of the synovial membrane may be an essential step towards fast pannus formation and consecutive destruction of adjacent cartilage. We hypothesize that the preferential presence of CD68+ cells towards the lining layer and the joint cavity with a rapid decline in the lower layers is due to an increase in extravasation of precursor cells from the blood, with more rapid homing towards the lining layer. Further evidence for this hypothesis is provided by the significantly higher expression of CD68, CD55, CD38 and CD3 in RA compared to OA which is in accordance with destructive pannus formation of RA being composed of macrophages, synovial fibroblasts, plasma cells, leucocytes and mast cells [28, 29].

In contrast to all other evaluated immune cells, CD3+ T cells did not have the tendency to accumulate in the upper sublining, but were distributed homogeneously. Depending on the inflammatory activity, CD3+ T cells were either absent, randomly distributed or clustered in follicle-like structures. These follicles, predominant in RA, spanned the entire sublining resulting in an intensive, but homogeneous staining pattern across all layers. Our description of different patterns is consistent with previous studies identifying and defining these histomorphological features in RA synovitis as ‘follicular’, ‘diffuse’ and ‘pauci-immune’ [30, 31].

Despite their inhomogeneous distribution patterns, we observed a moderate to high correlation of total CD68-, CD3- and CD55- staining in the entire sublining (i.e. not partitioned into different layers) and the synovitis score and its components, which has been established as a valuable tool to assess synovitis activity and to discriminate between low- and high-grade synovitis [17]. These data on one hand confirm CD68- expression as a valuable disease activity parameter and on the other hand prove the amount of sublining T cells and synovial fibroblasts to reflect the grade of synovitis and estimate disease activity. This again is supported by our finding of significantly higher expression of immune cell markers in RA, representing a more inflammatory phenotype [32] compared to OA.

There are some limitations to this study. Owing to the lack of any histological criteria clearly defining each layer, we divided the sublining into three zones of the same diameter which allowed us to directly compare results but did not consider interindividual differences regarding the extent of the sublining. We considered potential measurement inaccuracies rather minimal since ROIs were defined based on extensive study of all tissues and were set in similar areas adjacent to a straight lining layer with a sublining area of good tissue quality. To reduce intraindividual variations, three loci of each sample were analyzed. Since the patient selection was made according to clinical diagnosis only, without regarding other parameters like disease activity, duration of disease and medication due to ethical restrictions, the patient population was rather heterogeneous. In spite of that, results were consistent. Owing to our relatively small sample size, we did not further subclassify RA synovitis according to the aforementioned histological patterns [31, 33]. Furthermore, tissue obtained from either joint replacement or synovectomy implies a chronic or advanced state of disease. Future studies can assess cellular distribution within the synovial sublining employing linear mixed modelling in early disease states and its sensitivity to change following treatment. Hence, it has to be stressed that CD68 modelling is not yet fit for reliable diagnostic decision making until further diagnostic studies in early undifferentiated arthritis, including various inflammatory joint conditions, confirm our results in established RA. Moreover, although immune cell distribution is generally considered to be comparable between affected joints in polyarticular disease [34], we cannot fully exclude that differences observed reflect sample site rather than disease state.

Another limitation is that the semi-quantitative digital image analysis we applied, allowed a selection or deselection of single cells only to a limited extent through the thresholding step. CD38 can be present at low density in cells other than plasma cells like NK cells, B cells, T cells and macrophages so that in non-automated analyses usually only strong positive cells with the typical plasma cell morphology are counted [25]. We adjusted the threshold accordingly; nonetheless CD38 staining might be overestimated. Moreover, antibodies for immunohistochemistry typically represent the designated target cell, and are widely used for these purposes [35–37]. However, it should be noted that neither CD55 nor CD38 or CD68 are exclusively expressed by synovial fibroblasts, plasma cells, and macrophages [25, 38].

## Conclusions

Macrophages, synovial fibroblasts, plasma cells and mast cells show an inhomogeneous distribution within the synovial tissue in both RA and OA with highest concentrations in the upper sublining layer. Linear mixed modelling revealed a significantly higher concentration close to the lining layer with a more rapid decline in RA compared to OA. The model should be further analyzed for its performance as a biomarker and has pathophysiological implications.

## Abbreviations

ANOVA, analysis of variance; AUC, area under the curve; IQR, interquartile range; LMM, linear mixed model; MCT, mast cell tryptase; OA, osteoarthritis; RA, rheumatoid arthritis; ROC, receiver operating characteristics; SD, standard deviation; vWF, von Willebrand factor
